# Mock Juror Perceptions of Credibility and Culpability in an Autistic Defendant

**DOI:** 10.1007/s10803-018-3803-7

**Published:** 2018-10-31

**Authors:** Katie Maras, Imogen Marshall, Chloe Sands

**Affiliations:** 1 Centre for Applied Autism Research, University of Bath, Bath, UK; 2 Department of Psychology, Centre for Applied Autism Research, University of Bath, Claverton Down, Bath, BA2 7AY UK

**Keywords:** Autism, Defendant, Credibility, Culpability, Likeability, Honesty, Perceptions, Jurors, Criminal justice

## Abstract

One-hundred-and-sixty jury-eligible participants read a vignette describing a male who was brought to the attention of police for suspicious and aggressive behaviours and displayed atypical behaviours in court. Half of participants were informed that he had autism spectrum disorder (ASD) and were given background information about ASD; the other half received no diagnostic label or information. The provision of a label and information led to higher ratings of the defendant’s honesty and likeability, reduced blameworthiness, and resulted in fewer guilty verdicts, and more lenient sentencing. Thematic analysis revealed that participants in the label condition were more empathetic and attributed his behaviours to his ASD and mitigating factors, while participants in the *No label* condition perceived the defendant as deceitful, unremorseful, rude and aggressive.

Autism Spectrum Disorder (ASD) is a neurodevelopmental disorder characterised by impairments in social communication and interaction, and restricted and repetitive behaviours and interests (American Psychiatric Association 2013). For a small minority of individuals, characteristics linked to clinical features of the disorder may be associated with engagement with the police and criminal justice system (CJS) as an offender (e.g., Cheely et al. 2012; Heeramun et al. 2017; Helverschou et al. 2015; King and Murphy 2014; Lunsky et al. 2018; Mouridsen 2012; Rava et al. 2017; Tint et al. 2017; Turcotte et al. 2017; Vohra et al. 2016; Woodbury-Smith and Dein 2014). 1 For example, obsessional or circumscribed interests and behaviours (e.g., Barry-Walsh and Mullen 2004; Chen et al. 2003; Hare et al. 1999; Haskins and Silva 2006; Helverschou et al. 2015; Woodbury-Smith et al. 2010), misinterpretation of rules (Allen et al. 2008), failing to recognise the consequences of one’s actions (Howlin 2004), interpersonal naivety and poor social awareness (Haskins and Silva 2006; Murrie et al. 2002), idiosyncratic interpretation of people and events (Helverschou et al. 2015; Katz and Zemishlany 2006; Woodbury-Smith et al. 2005), challenging behaviours (Tint et al. 2017), sensory sensitivities (Katz and Zemishlany 2006; Mawson et al. 1985) and difficulties relating to empathy (Bjørkly 2009; Murrie et al. 2002), emotion recognition (Woodbury-Smith et al. 2005), impulse control and emotional dysregulation (Lerner et al. 2012) have all been implicated in various cases of autistic offending.

For some individuals, precipitating factors such as family issues, a deterioration in mental health, disruption to routine, stress, or sensory overload (Allen et al. 2008; Helverschou et al. 2018; Mouridsen et al. 2008) may act on predisposing factors such as social naivete´, lack of awareness over consequences of one’s actions, impulsivity, or obsessional interests to result in offences such as unintentional acts of aggression (Cheely et al. 2012; Helverschou et al. 2015; Lerner et al. 2012; Tint et al. 2017; but see; Ghaziuddin et al. 1991). For example, Allen et al. (2008) describe qualitative findings from a small sample of autistic offenders indicating that violent or destructive behaviours were almost always precipitated by an accumulation of stress, exacerbated by maladaptive coping strategies. Tint et al. (2017) followed a large sample of autistic youth and adults over a 12–18-month period and reported that 16% had some form of police involvement during this time, with physical or verbal aggression towards others in the community the most frequent reason for contact (e.g., threatening to harm a teacher at school). It is important to note, however, that co-occurring psychiatric conditions also play a role in many cases of violent offending in ASD (Allen et al. 2008; Haw et al. 2013; Helverschou et al. 2015; Långström et al. 2009; Newman and Ghaziuddin 2008). Indeed, a large population-based study, Heeramun et al. (2017) found that ASD actually served as a protective factor for violent offending once other co-occurring diagnoses such as ADHD were accounted for (see also Lundström et al. 2014).

It is important to understand how legal professionals, jurors and other decision makers within the CJS perceive autistic individuals and whether ASD-related factors are taken into account in judgements about their offending behaviours. Information on ASD is sometimes used in court during sentencing (Freckelton and List 2009), yet the impact of this on juror perceptions and judgements about the defendant is currently unclear and is likely to depend to some extent on how the condition and the individual are perceived (Allely and Cooper 2017; Berryessa 2016). Some evidence suggests that an ASD diagnosis may have little impact on decisions of criminal responsibility (Freckelton and List 2009; Helverschou et al. 2015), while others have reported that judges may take ASD into account as a mitigating factor (Berryessa 2014) or that an autistic offender may be less likely to receive a custodial sentence and instead diverted out of the CJS (Allen et al. 2008). However, it has also been reported that legal professionals struggle to determine what emphasis to place on diagnosis and other information from psychiatric reports (Berryessa 2014), and while some use it as a mitigating factor, others consider ASD to be an aggravating factor (Berryessa 2016).

The effect that the presentation of information about an individual’s diagnosis has on determining responsibility in court needs thorough consideration (Allely and Cooper 2017; Freckelton 2011). In the only study to date examining juror perceptions of autistic defendants, Berryessa et al. (2015) utilised a within-subjects survey design to ask lay participants about their perceptions of a hypothetical autistic defendant before and after they were informed that the defendant had an ASD diagnosis. Findings suggest that participants were more sympathetic and positive once they knew that the defendant had ASD and this mitigated their judgments of moral responsibility and criminal intention, but it did not affect their views of legal responsibility or criminality. However, the repeated measures design used in this study makes it difficult to rule out alternative explanations such as carry-over effects, response shift biases, and demand characteristics.

An autistic person may also be viewed strange or awkward in court, which is likely to have further repercussions for the judgements that are made against them. Observers often base their judgments of an individual’s credibility on verbal and non-verbal cues (DePaulo et al. 2003) such as eye-contact (Hartwig and Bond 2011), body movements and fidgeting (Strömwall and Granhag 2003), surface features of speech (Ozuru and Hirst 2006) and displays of emotion (Tallon et al. 2015). ASD manifestations including atypical eye contact (Neumann et al. 2006; Senju and Johnson 2009), unusual gestures and stereotyped body movements (de Marchena and Eigsti 2010; Gritti et al. 2003), atypical speech characteristics and prosody (Peppe ´ et al. 2007), and unusual expression of emotion (Loveland et al. 1994) may all, therefore, have potentially serious and negative consequences (Cea 2014; Tsoudis 2000). Indeed, recent research shows that atypical behaviours often result in negative impressions and judgements about autistic individuals in everyday contexts (Faso et al. 2015; Grossman 2015; Grossman et al. 2018; Harnum et al. 2007; Sasson et al. 2017; Sasson and Morrison 2017). Critically, however, while first impressions of autistic adults are often less favourable, several studies have recently reported that informing observers of their ASD diagnosis results in significantly more favourable judgements about them (Brosnan and Mills 2016; Butler and Gillis 2011; Matthews et al. 2015; Sasson and Morrison 2017).

While there is therefore some limited evidence to suggest that providing a diagnosis label may result in more positive attributions and judgements about an autistic defendant, there is often a reluctance among some autistic individuals to disclose their diagnosis in the CJS due to concerns about a lack of autism knowledge and awareness, and fear of discrimination or victimisation by others (Crane et al. 2016; see also; Shtayermman 2009). This may not be entirely unfounded given that negative media portrayals of an association between ASD and violent and criminal behaviours have been shown to result in negative attitudes towards autistic individuals (Brewer et al. 2017), and there is concern that juries make decisions based on stigmatized beliefs and misconceptions of ASD and other clinical groups (Allely and Cooper 2017; Blais and Forth 2014; Boccaccini et al. 2008; Jochnowitz 2011; Mayes 2003). However, knowledge and attitudes about ASD have improved in recent years (Dillenburger et al. 2013; White et al. 2016), and in turn knowledge has been shown to decrease stigma (Gillespie-Lynch et al. 2015). Recently, Crane et al. (2018) reported that providing a diagnosis label alongside further generic information about ASD resulted in more positive credibility ratings of an autistic child witness. Interestingly, however, this effect was not observed for a second autistic child witness who displayed fewer atypical behaviours, highlighting the impact of heterogeneity in the presentation of autistic behaviours and potentially a need for tailored, individualised information about how ASD affects that particular person. Within the CJS, the provision of such information could have a profound effect on the treatment an individual receives and legal decisions that are made about them.

The aim of the present study was to examine mock juror perceptions of credibility and culpability of a defendant who is described as displaying autistic-like characteristics and behaviours, and whether the provision of information about the defendant’s ASD diagnosis alters these perceptions. Participants read a vignette describing a male who was brought to the attention of police for suspicious and aggressive behaviours and displayed unusual behaviours once in court. Half of participants were informed that he had a diagnosis of ASD and read a report from a forensic psychiatrist about what ASD is and how the defendant was affected by it; the other half received no diagnosis label or further information. Participants then rated his credibility and culpability and provided qualitative justifications for their ratings. Using a mixed-method approach, we aimed to gain a more in-depth understanding of the reasoning behind jurors’ decisions to these questions (Trahan and Stewart 2013).

Based on research suggesting that a label and knowledge can provide an alternative explanation for atypical behaviours and diminish negative stereotypes (e.g., Brosnan and Mills 2016; Butler and Gillis 2011; Crane et al. 2018; Gillespie-Lynch et al. 2015; Matthews et al. 2015; Sasson and Morrison 2017), it was predicted that providing an ASD label and further information about the condition would lead to higher ratings of credibility. We also tentatively predicted that a diagnosis label and further information would result in the defendant being perceived as less culpable for their actions, with less perceived blameworthiness, fewer guilty verdicts and more lenient sentencing attitudes towards him.

## Method

### Design

To avoid carryover effects, the study employed a between-participants survey design whereby mock jurors were randomly assigned to one of two label conditions: ‘*Label*+*info’*, in which mock jurors were informed that the defendant was autistic and were given further information about the condition and how the individual was affected by it; or *‘No label’* in which no diagnosis or information about ASD was provided. The dependent variables were mock juror ratings of the defendant’s credibility, in terms of likability, cognitive functioning, and honesty (on a 1–7 Likert scale), as well as perceived blameworthiness (1–7 scale), guilty verdict (guilty, not guilty) and sentencing leniency (1–7 scale). Participants’ responses to follow-up open ended questions formed the basis of a qualitative analysis of the reasons for their ratings and judgements.

### Participants

An opportunity sample comprising 160 participants who were eligible for jury service in the UK (i.e., aged 18–75, not lacking capacity within the meaning of the Mental Capacity Act, and not recently serving criminal convictions) participated in this study. Participants were recruited via advertisements placed on social media (e.g., Facebook, Twitter), local volunteer organisations, and the Research Community Participation Panel at the University of Bath. As an incentive for taking part, participants were invited to enter a prize draw to win £50 in Amazon vouchers. Participants ranged in age from 18 to 68 years (*M* = 28.17 years, *SD* = 12.93), and comprised 42 males and 118 females. The majority of participants were employed (55.6%), and/or currently studying (61.9%), with 20.6% having studied psychology/ psychiatry to degree level or higher. Participants were randomly assigned to either the *Label*+*info* (n = 80; mean age = 29.70, *SD* = 13.68; 22 males) or *No label* condition (n = 80; mean age = 26.66, *SD* = 12.03; 20 males). Ethical approval was obtained from the University of Bath’s Psychology Research Ethics Committee and all participants provided their informed consent to take part.

### Materials and Procedure

All participants completed the study on the online Qualtrics data system (http://www.qualtrics.com).

#### Condition Manipulation

Prior to reading the vignette, respondents in the *Label*+*info* condition were informed that the defendant had been assessed by a forensic psychiatrist and was diagnosed with ASD, and they were given further information defining ASD and how it impacted on his behaviours. For example, participants were told the defendant sometimes found it difficult to communicate appropriately, experienced sensory sensitivity, and often felt highly anxious in unfamiliar situations (see “Appendix 1”). For the *No label* condition, respondents received no diagnosis or further information prior to presentation of the vignette.

#### Vignette

The vignette described a 27-year-old male who was arrested and appeared in court on a charge of assault and battery of a police officer. It comprised two main sections: ‘The Case Summary’ and ‘At Court’ (see “Appendix 2”). The Case Summary contained background information of the offence; participants were informed that the defendant was behaving aggressively at a train station and when police officers tried to restrain him with handcuffs, he became violent and struck an officer. During the police interview it reportedly emerged that the defendant was trainspotting and was upset because his train was cancelled. The At Court section provided a short excerpt of the prosecutor questioning the defendant in court. The defendant’s crime and behaviour in court was portrayed in a manner consistent with ASD symptomatology and previous research on offending in ASD. This included aggressive behaviours that were reactive to high stress, invasion of personal space and disruption to routine (Allen et al. 2008; Cea 2014; Freckelton and List 2009; Kanne and Mazurek 2011; Mouridsen et al. 2008; Tint et al. 2017), and brought about following the defendant’s pursuit of his circumscribed interest in trains (e.g., Barry-Walsh and Mullen 2004; Chen et al. 2003; Hare et al. 1999; Haskins and Silva 2006; Helverschou et al. 2015; Woodbury-Smith et al. 2010). The defendant also displayed high levels of anxiety and intolerance of uncertainty (Wigham et al. 2015) alongside impaired social-communication, poor eye contact, sensory sensitivities, restrictive and repetitive behaviours, and a need for sameness (American Psychiatric Association 2013).

#### Credibility and Culpability Judgements

After reading the vignette participants were asked to rate (on a series of 7-point Likert scales) their perceptions of the defendant’s cognitive functioning, honesty, and likability. Participants were also asked to what extent they believed the defendant was to blame for the incident (7-point Likert scale), whether they felt he should receive a guilty or not guilty verdict (guilty, not guilty) and, if they reported that he should be found guilty, how harshly he should be sentenced (7-point Likert scale). These questions were adapted for the purposes of the current study aims from previous mock juror research examining credibility (Henry et al. 2011; Mueller-Johnson et al. 2007), and blameworthiness, guilt and sentencing leniency (Mowle et al. 2016; Wiley and Bottoms 2009). Following each rating participants were asked to provide qualitative responses regarding why they gave that rating (e.g., ‘Why did you give this rating of likeability? Please explain your answer’).

### Analysis Plan

First, in order to examine whether the provision of an ASD label and further information impacted juror perceptions of credibility, a one-way multivariate analysis of variance (MANOVA) with separate univariate tests was run with the three credibility variables: cognitive functioning, honesty, and likability. Next, analyses examined mock juror perceptions of culpability and sentencing. A *t* test was used to examine the effect of diagnosis label on mock juror ratings of blameworthiness, and a Chi square test of independence tested for an association between the provision of an ASD label and guilty versus not guilty verdicts. For participants who provided a guilty judgment, a second independent samples t-test was run to determine if there were differences in their ratings of sentencing leniency between the *Label*+*info* condition and the *No label* condition.

Finally, participants’ responses to the open-ended questions were qualitatively analysed by the second author (IM) using an inductive thematic analysis, whereby themes were data-driven and emerged naturally from the data (Braun and Clarke 2006). Thematic analysis was chosen on the basis that it is dynamic, flexible and suitable for large data sets (Agee 2009). The data corpus was analysed as a whole; however, because participants were responding to questions that asked them to justify their rating scores their responses were naturally organised around each of the question themes, and therefore the codes that emerged often tended to centre on these themes. The analysis was conducted according to Braun and Clarke’s (2006) six recommended stages: familiarisation with the data (reading and noting down initial ideas); generating initial codes in a systematic fashion across the entire dataset (collating data relevant to each code); searching for themes (collating codes into potential themes and gathering all data relevant to each potential theme); reviewing themes (at both the level of the extracts and across the entire dataset) and generating a thematic map of the analysis; defining and naming themes (refining the specifics and definitions of each theme and the overall story of the analysis); and reporting what was found. This allowed a more detailed and nuanced examination of mock-jurors’ perceptions and judgements, to complement the quantitative analyses and extract deeper meaning from the data. Analyses were also independently conducted and reviewed by CS, and all authors reviewed and resolved discrepancies before agreement was reached on four overarching themes with several subthemes within each of these.

## Results

### Credibility

A one-way MANOVA revealed a significant multivariate effect of label condition on the three combined credibility dependent variables, Pillai’s Trace = 0.11, *F*(3, 156) = 6.31, *p* < .001, ηp^2^ = .11. Univariate tests showed that honesty, *F*(1, 158) = 14.96, *p* < .001, ηp^2^ = .09, and likability, *F*(1, 158) = 7.47, *p* = .007, ηp^2^ = .05, were significantly higher for participants in the *Label*+*info* condition compared to those in the *No label* condition. There was no significant effect of condition on ratings of cognitive functioning, *F*(1, 158) = 0.90, *p* = .411, ηp^2^ = .004. Table 1 summarises these data.

**Table 1:** Mean ratings of the defendant’s credibility (cognitive functioning, honesty and likeability) and blameworthiness by participants in the *Label*+*info* and *No label* conditions (standard deviations are in parentheses)

	*Label*+*info*	*No label*
Cognitive functioning	3.68 (1.17)	3.53 (1.14)
Honesty*	5.93 (1.08)	5.13 (1.50)
Likability*	3.30 (1.13)	2.79 (1.24)
Blameworthiness*	3.66 (1.41)	4.73 (1.33)

*Significant between condition difference, *p* < .001

### Culpability and Sentencing

**Fig. 1:**
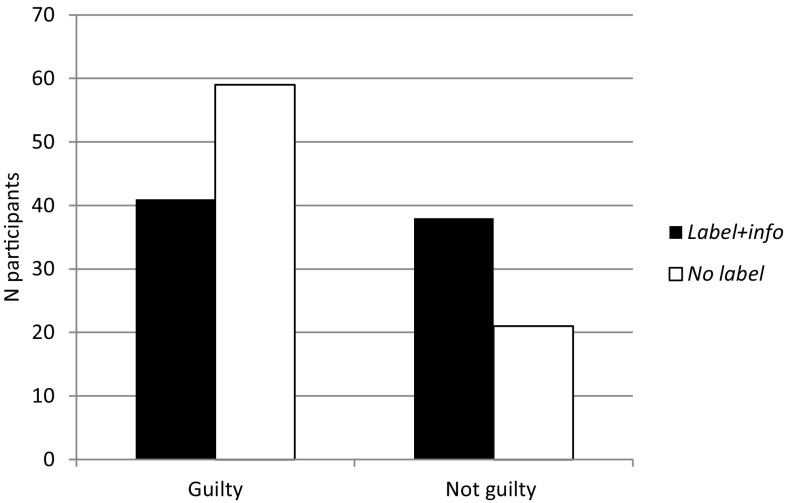
Judgements of whether the defendant should receive a guilty or not guilty verdict by participants in each label condition

Participants in the *Label*+*info* condition reported feeling that the defendant was less to blame for his actions than participants in the *No label* condition, *t*(158) = 4.89, *p* < .001, Cohen’s *d* = 0.78 (Table 1), and there was a significant association between the information that mock jurors received about the defendant’s diagnosis and their beliefs regarding whether he should receive a guilty or not guilty judgement, *χ*2 (1, *N* = 159) = 8.13, *p* = .004. Participants in the *No label* condition were more likely than participants in the *Label*+*info* condition to assign a guilty verdict, while participants in the *Label*+*info* condition were more likely than participants in the *No label* condition to assign a not guilty verdict (Fig. 1).

Participants who assigned the defendant a guilty verdict were asked how harshly they felt he should be sentenced. Participants in the *Label*+*info* condition reported more lenient sentencing attitudes towards the defendant (*M* = 2.37, *SD* = 0.94) than participants in the *No label* condition (*M* = 2.83, *SD* = 1.10), *t*(98) = 2.20, *p* = .030, Cohen’s *d* = 0.45.

### Reponses to Open Questions: Thematic Analysis

Four overarching themes emerged from the data corpus as a whole: honesty, inappropriate language, culpability, and consequences, respectively. Further *subthemes* were identified within each of these main themes, which are outlined below and detailed in Table 2 alongside example quotes.

**Table 2:** Themes and subthemes identified from the qualitative data, with example quotes

Theme	Subtheme	Example quotes
Honesty	Excessive candour	“He was very frank about his opinion of his coffee, even telling the lawyer his aftershave was unpleasant suggesting a lack of restraint with brutal honesty.” (P121, *Label*+*info*) “He doesn’t appear to try and deceive anyone and if anything seems overly honest e.g. in relation to lawyer’s aftershave.” (P137, *No label*) “he wasn’t trying to hide anything” (P128, *Label*+*info*)
	Autistic people cannot lie	“Generally people with ASD do not lie…” (P38, *Label*+*info*) “…Knowing the facts about his autism I assumed he would not have the cognitive ability to dissemble or lie about facts…” (P74, *Label*+*info*)
	Dishonesty	“Remembers the quality of coffee very well yet cannot remember hitting the police officer. To me this shows his ability to lie or hide information for his own interests”. (P97, *No label*) “His body language of avoiding eye contact suggests dishonesty and his aggressive language comes across as overly defensive.” (P119, *No label*)
Inappropriate Language	Sympathetic due to ASD	“Obviously due to his ASD he likely finds it a bit difficult to act in a way that others might deem appropriate for court, so he comes across as rude ... It sounds as if the whole situation was brought about just by stress. … So he’s probably a bit rude but that might be due to his circumstances currently rather than his general persona.” (P151, *Label*+*info*) “Due to his ASD, Mr Parsons can be very direct and even rude (for example, commenting on the overpowering aftershave), therefore he does not come across as very amiable. However, I do not believe at all that this is his intention.” (P160, *Label*+*info*) “He is very blunt and unengaged. He is quite abrupt with the lawyer about the aftershave and could appear rude. However given the knowledge of his autism his sensory and social struggles do evoke empathy.” (P146, *Label*+*info*)
	Negative, compounded by other factors	“He swore, he was rude about the aftershave, he showed little remorse.” (P55, *No label*) “He was rude to the lawyer, using bad language and avoiding eye contact.” (P84, *No label*) “He acts disrespectfully and unremorseful, especially considering the circumstances where he is likely to be prosecuted for his actions.” (P119, *No label*)
Culpability	Mitigating factors/diminished responsibility	“It is doubtful he is often aggressive, but the events that started with missing his train and ruining his routine may have caused irregular behaviour due to his ASD.” (P36, *Label*+*info*) “I think if the police officers understood ASD more the situation could have been handled better and Mr Parsons wouldn’t have become as aggressive. His disorder caused him to become anxious and then the police officers made him even more anxious causing him to lash out but I don’t think he did it out of spite or because he is a violent person, it’s because he lost control after getting upset.” (P144, *Label*+*info*) “Mr Parsons if he can stick to his usual routine will never be a problem I feel it was other people’s perception of his behaviour and the police officers total misunderstanding of both his illness and the situation which were mainly to blame.” (P1, *Label*+*info*) “I suppose it is society that is to blame, we don’t know how to handle those who are different.” (P101, *Label*+*info*) “Not guilty—Firstly, I considered whether he actually did it (which he clearly did). However, I then considered whether he could be held responsible (arguably not, due to the circumstances it may be considered that he had diminished responsibility) and whether he intended harm (again, I do not believe he did).…I judged the factors of responsibility (whether he was to blame) and intent, to be the most important.” (P160, *Label*+*info*) “I don’t believe that his cognitive function is high enough to say that he had enough control over himself to prevent what happened in any way. I don’t feel 100% comfortable with this decision, and if the defendant wasn’t autistic it would be easy to decide that he was guilty. … I also think that the police officers involved should have been able to spot that he was autistic during the incident and reacted accordingly rather than trying to deal with him as they would with anyone else.” (P5, *Label*+*info*) “Not guilty—It was in no way intentional, he was distressed and uncomfortable.” (P61, *No label*)
	Aggravating factors/responsible for actions	“He’s got social inadequacies, so the situation set him off and the police made it worse, but he isn’t mentally incapable so ought to be able to take responsibility for coping with his own reactions to adverse circumstances. He’s not a child anymore.” (P37, *No label*) “Seems to have a short fuse and aggressive tendencies.” (P137, *No label*) “Because he wanted to behave in this manner because he doesn’t care.” (P125, *No label*) “The defendant was unprovoked by another human and had an irrational reaction to an everyday occurrence.” (P116, *No label*) “I rated him as a 7…we have not been told is mentally ill or sick so to my knowledge he is definitely to be blamed for the incident.” (P58, *No label*) “Guilty—He has an attitude of not having respect for anything.” (P125, *No label*) “Guilty—His lack of cooperation, explanation, and remorse contribute to my decision but most importantly are multiple witnesses at the station and clear evidence of the officer who was struck regardless of the intention.” (P119, *No label*) “Guilty—The violence in which he portrayed, during and after the crime (in the police station) and the image that he portrayed of himself in court.” (P67, *No label*)
Consequences	Punishment	“…it’s important to show society that he didn’t just get away with it by giving him some form of punishment.” (P83, *No label*) “After all the crime was for assaulting a police officer and he did do it—so he is guilty. The punishment however I feel should reflect the incident in itself, such like community service rather than sentence to prison.” (P127, *No label*) “He was only being disruptive, it happens, a small punishment would be enough” (P2, *No label*) “His condition would be compounded by harsh punishment.” (P23, ASD+label)
	Doubt	“He didn’t mean to hit the police officer but that doesn’t take away from the fact that he did so I’m not confident in my choice because I’m not really sure how compassionate the judicial system is.” (P159, *No label*) “I think the court will rule him guilty because they know that he did punch the police officer, I am not confident in it because I do not think that Mr Parsons should be punished for it.” (P139, *No label*) “He did assault the officer.—most important. But I don’t know much about the law and as I mentioned I don’t know whether the police officers acted correctly at the scene and I don’t know if it was an accident (because he said he didn’t mean to but that could be that he was flailing or it could be that he aimed to hit the guy at the time but didn’t think it through just reacted).” (P114, *Label*+*info*)
	Rehabilitation	“I also considered whether a guilty verdict would be of benefit to Mr Parsons and society. I do not believe it would be, and I believe help and rehabilitation for Mr Parsons would be of greater benefit.” (P160, *Label*+*info*) “I would also consider the consequences of a guilty verdict—it wouldn’t seem sensible to send this man to prison… perhaps there might be an appropriate psychological intervention.” (P66, *Label*+*info*) “I don’t believe the sentence will help him, he needs help from professionals” (P83, *No label*)

#### Theme 1: Honesty

The defendant was generally viewed as very honest, with many participants referring to his comments in court (e.g., “even telling the lawyer his aftershave was unpleasant”) as evidence of this. While many participants were sympathetic to what they viewed as *excessive candour*, describing it is as positive attribute, others viewed it as aggressive and inappropriate. There was also a belief by many participants in the *Label*+*info* condition that *autistic people cannot lie*, which led mostly to them viewing the defendant more positively. However, in the *No label* condition, while some participants viewed the defendant as generally honest, others felt that his behaviour (e.g., “avoiding eye contact”) and “aggressive” language were actually indicative of *dishonesty* and that he was lying in order to protect himself.

#### Theme 2: Inappropriate Language

The majority of participants perceived the defendant’s swearing and language towards to the lawyer as off-putting and rude. This was framed sympathetically by some (e.g., “due to his circumstances”) but negatively by others, resulting in two subthemes. The majority of participants in the *Label*+*info* condition acknowledged the defendant’s language as rude but were more *sympathetic due to his ASD* and made allowances for this. However, participants in the *No label* condition felt that the defendant’s inappropriate language was *compounded by other factors* which resulted in negative perceptions. For example, his apparent lack of remorse, avoiding eye contact and swearing were frequently referred to by participants as “unhelpful”, “off-putting” and “rude”.

#### Theme 3: Culpability

Participants in the *Label*+*info* condition predominantly focused on *mitigating factors* (elements of the case which reduced the defendant’s responsibility or culpability), such as a lack of intent and the defendant’s ASD. They acknowledged the defendant’s crime, but suggested it was the result of his ASD and the situation he was in. Specifically, they felt that his ASD caused him to become easily “overwhelmed” and “anxious”, and that his actions were “in no way intentional” but a reactive response to a stressful situation, and that a lack of control over this made him less blameworthy. Several participants also questioned whether the officers acted appropriately and had been trained to deal with this kind of situation, thus transferring some of the blame to the actions of the police officers.

Participants in the *No label* condition, in contrast, focussed more on *aggravating factors* which increased the defendant’s culpability, such as the defendant having a “short fuse and aggressive tendencies”, or his apparent lack of concern and respect for others. While some participants in the *No label* condition also acknowledged situational factors, these participants nevertheless felt that the defendant should “take responsibility for coping with his own reactions”.

#### Theme 4: Consequences

A number of participants in both conditions considered the consequences of a guilty verdict when deciding responsibility. While some participants in the *No label* condition felt that *punishment* was necessary “to show society that he didn’t just get away with it”, most felt that the punishment should be small given the nature of the crime. A common concern, particularly among those in the *Label*+*info* condition, was that the punishment associated with finding the defendant guilty would be of little benefit. Indeed, many participants expressed *doubt* relating to knowing that the defendant was guilty yet lacking confidence in assigning a guilty verdict and feeling concerned over how this verdict and the associated punishment would affect the defendant. Many participants (particularly in the *Label*+*info* condition) suggested that some form of *rehabilitation* to help the defendant would be more appropriate than a prison sentence. Although to a lesser extent, some participants in the *No label* condition also recognised that the defendant needed help “from professionals”.

On completing the study participants in in the *No Label* condition were asked they thought the defendant had a developmental disability or mental health condition. More than half (57.5% n = 46) of participants in this condition indicated that they thought the defendant might have ASD.

## Discussion

The present study found that a defendant who was described as exhibiting autistic-like behaviours was perceived as more credible and less culpable for his actions if information about his ASD diagnosis was known. Specifically, mock juror participants who were provided information about the defendant having a diagnosis of ASD and how he was affected by it (‘*Label*+*info’*) rated him as more honest and likeable, and less blameworthy for his actions. They were also more likely to suggest a not guilty verdict and were more lenient in their views on appropriate sentencing, compared to participants who received no information about his diagnosis (‘*No label*’). In fact, the only variable on which there was no significant difference between label conditions was for perceptions of the defendant’s level of cognitive functioning.

Qualitative analysis of participants’ justifications for these ratings indicated that knowledge of the defendant’s diagnosis resulted in more empathetic feelings towards him, with perceptions of excessive candour (due to his ASD), more sympathetic views of his inappropriate language and behaviour (which was viewed as unintentional and uncontrollable), a greater focus on mitigating factors (e.g., to his ASD, the situation, and a perceived lack of underlying intention behind his actions) and the consequences of being found guilty when assigning him a not guilty verdict. In contrast, participants in the *No label* condition reported that the defendant’s aggressive behaviours, evasive answers, inappropriate language towards the lawyer, and lack of apparent remorse exacerbated their negative perceptions and judgements of culpability and guilt. This is consistent with previous research showing that remorse is also a significant factor in sentencing decisions by both mock jurors and judges (MacLin et al. 2009; Wood and MacMartin 2007; see also; Berryessa et al. 2015), and supports Haskins and Silva’s (2006) contention that special considerations should be made for autistic defendants who are unable to express remorse (see also Lerner et al. 2012). It is pertinent that these negative perceptions and attributions were reported despite more than half of participants in the *No label* condition indicating they thought the defendant might have ASD, suggesting that the suspicion of ASD was not enough to influence their judgements; a label was necessary to mediate improved credibility ratings and mitigate responsibility and guilt.

Some participants in the *Label*+*info* condition nevertheless expressed uncertainty over the extent to which the defendant’s ASD should act as a mitigating factor for guilt. For example, one participant reported that “*if the defendant wasn’t autistic it would be easy to decide that he was guilty*”. Establishing responsibility for an autistic defendant may be harder than for other groups (Allely and Cooper 2017; Byrd 2018), and whether an autistic defendant has the capacity to form intent is a complex issue requiring careful consideration (Woodbury-Smith and Dein 2014; see also; Blair 2001; Cea 2014; Hall 2015; Freckelton and List 2009; Mayes 2003). Jurors often make attributions based on the perceived controllability of behaviour (Weiner 1985, 2006) and conditions that are rooted in biological bases are often considered uncontrollable and therefore mitigating factors (e.g., Aspinwall et al. 2012). This has been shown to result in perceptions of diminished blameworthiness and culpability in other groups such as those with intellectual disabilities and schizophrenia (Alicke 1990; Barnett et al. 2007; Bottoms et al. 2003; Mossière and Maeder 2016; Najdowski et al. 2009).

The current findings that the provision of an ASD label impacted perceptions of guilt, even though the case itself was unambiguous (it was clear that an assault was committed), provides a further striking example of the influence that a diagnosis can have on perceived culpability, raising important legal, philosophical, and ethical questions including whether ASD could and should be considered a “get out of jail free card” (Sutton 2014). The present findings indicate that providing information about ASD is useful to prevent negative perceptions of autistic behaviours, however further research needs to extend the current findings to explore whether it results in jury members being unduly lenient and to what extent this information should be considered in terms of mitigation. Nevertheless, it is important to note that an ASD diagnosis should, at least, be used to lead to reasonable adjustments to ensure fair access to trial, for example with the provision of an intermediary to give evidence (Cooper and Allely 2017), and there is also a requirement for assistance to be given to juries to make decisions informed by expert insights of the needs and complexities of ASD (Allely and Cooper 2017; Freckelton 2013).

Participants’ qualitative responses to the follow-up questions indicated that higher sentencing leniency and more not guilty verdicts in the *Label*+*info* condition were also related to considerations regarding the defendant’s ability to cope with the CJS. This is consistent with previous research suggesting that, given potentially damaging effects of prison on an autistic individual, both lay persons and judges often view rehabilitation methods as preferable to harsher prison sentences (Berryessa 2016; Berryessa et al. 2015). Moreover, the purpose of imprisonment is to prevent future crimes, yet if an individual commits a crime due to factors that are largely outside of their control, such as sensory overload or a lack of awareness of the situation, prison is unlikely to be a deterrent for committing future offences. Evidently, the relationship between providing an ASD label and perceptions of culpability is convoluted, with several routes towards reducing blame (see also Wasserman et al. 2010).

The present findings that the provision of an ASD label together with background information about the disorder resulted in more positive likeability and honesty perceptions of an autistic defendant is in line with previous findings in everyday contexts as well as for child witnesses (Brosnan and Mills 2016; Crane et al. 2018; Matthews et al. 2015; Sasson and Morrison 2017; Sasson et al. 2017). In contrast, the defendant’s lack of remorse troubled many participants in the *No label* condition who took it to imply that he was disinterested, uncaring, and did not regret his actions, which reduced his overall likeability. On the whole, participants also did not like the defendant’s swearing and rudeness, but those in the *Label*+*info* condition were more sympathetic and viewed it as a by-product of his ASD rather than an inherent personality characteristic, thus moderating the diminution of his likeability. Previous research examining first impressions of autistic individuals suggests that how an individual presents themselves socially drives impression formation, with autistic individuals often being perceived more negatively in this context (e.g., Sasson et al. 2017; Sasson and Morrison 2017). Consistent with this, qualitative responses in the present study highlighted that when no diagnosis label was provided, respondents often reported characteristics of autism as aggravating factors that made the defendant seem less likable.

Participants in the *Label*+*info* condition also reported a strong assumption that autistic individuals cannot or do not lie, which is only partially supported by a limited and inconsistent body of literature with children (e.g., Li et al. 2011; Talwar et al. 2012; Yang et al. 2017). In contrast, participants in the *No label* condition perceived the defendant as a character who was lying for his own interests, based on his body language, difficulty recalling the event, and lack of eye contact. This is consistent with research showing that credibility assessments are often based on verbal and non-verbal behaviours such as eye contact (Wright and Wheatcroft 2017) twitchy and repetitive movements (e.g., Granhag et al. 2004), demeanor (Levine et al. 2011), and story-telling ability (DePaulo et al. 2003).

Recent findings suggest that many autistic individuals are reluctant to disclose their diagnosis for fear of discrimination and a lack of understanding. For example, Crane et al. (2016) found that 36% of individuals with ASD who had CJS contact never disclosed their diagnosis for fear of victimisation and discrimination, and Maras et al. (2017) reported that 92% of solicitors and barristers questioned had experienced cases where the defendant’s diagnosis was not disclosed until trial. To the contrary, the present findings add to a small but recently accumulating body of literature suggesting that revealing a diagnosis of ASD largely results in more positive perceptions. These findings have implications for the CJS and beyond. Disclosing one’s ASD diagnosis in the workplace or at school, for example, may help others understand the challenges they face, increase understanding and explain any unusual or inappropriate behaviour. This might motivate autistic individuals to disclose their diagnosis in both formal contexts such as the CJS and in more social situations such as when meeting new people or trying to form friendships. Future research should now extend this to other contexts such as employment interviews.

Limitations of the present study are acknowledged. First, while more representative than a psychology student sample, one-fifth of the current participants had studied psychology and the majority were white and female. Nevertheless, that more than half of those in the *No label* condition guessed that the defendant might have ASD, yet still significant differences emerged suggests that if anything the present study may have underestimated the effect that a label had on juror perceptions. A second limitation is that the study design did not incorporate juror instructions, such as how to assess evidence and how to determine credibility, which may have been why several participants articulated uncertainty and doubt over their decision. Third, while vignettes are one of the most commonly used methods in establishing attitudes towards certain vulnerable groups (Swami 2012) they do not allow for participants to view or hear idiosyncratic aspects of a live testimony. The present study nevertheless provides an important step to understanding the basic perceptions of jurors on autistic defendants. Following this, there is scope to examine perceptions in a more naturalistic setting. Future research may wish to consider how mock jurors make decisions based on videos of autistic defendants’ testimonies.

Heterogeneity within cases, offences and offenders also highlights the need for future research to replicate and extend this study with different methodologies. Future research should explore, for example, perceptions of defendants committing different types of offences, such as those involving sexual assault, stalking, or hacking. Given the high rate of co-occurring diagnoses such as ADHD that have been suggested to underlie certain types of offending behaviour in ASD (Allen et al. 2008; Haw et al. 2013; Helverschou et al. 2015; Långström et al. 2009) it is also important to examine the impact of providing other types of labels on juror perceptions.

## Conclusion

The present study aimed to examine whether disclosure of a defendant’s ASD impacted mock-juror judgements of credibility and culpability. Findings suggest that the information provided to jurors about an autistic defendant can have a profound effect on their judgements of his likeability, honesty, blameworthiness, guilt and sentencing. With the provision of an ASD diagnosis label and further information, the defendant’s ASD was predominantly seen as the uncontrollable cause of his behaviour, compounded by the situation he was in. This has important implications not only for the courts, but also for other areas such as employment and social situations where the disclosure of an individual’s ASD diagnosis may impact others’ perceptions of them. While the present findings suggest that revealing a diagnosis of ASD could be beneficial, existing literature suggests autistic individuals are fearful of doing this. However, the present findings indicate that autistic individuals who do not reveal their disorder may be perceived more negatively, while an ASD label motivates compassion, understanding and mitigates responsibility. These findings have significant implications for disclosure and should be an encouragement to the ASD community.

## Appendix 1: Background Provided to Participants in the *Label*+*info* condition

This study concerns a 27-year-old male defendant, in court on a charge of assault and battery of a police officer. Full name: Mr. David Alan Parsons.

Mr. Parsons has been assessed by a forensic psychiatrist and has been diagnosed as having Autism Spectrum Disorder (ASD), which is defined by problems with social communication and interaction, and repetitive behaviour patterns and restricted interests (American Psychiatric Association 2013).

Mr Parsons has difficulty forming relationships with peers, and therefore spends a lot of time on his own and doesn’t have many friends. He is socially awkward and struggles to communicate with others appropriately; for example, sometimes his facial expressions and tone of voice are inappropriate for the social context, and he struggles to understand sarcasm and irony. Mr Parsons also demonstrates repetitive behaviour patterns and has a rigid adherence to routines, which he can get extremely anxious about if he is not able to follow. Unfamiliar situations can also cause a high degree of anxiety that have been known to result in aggression. Mr Parsons has a narrow range of specialist interests, including a preoccupation with trains, and also experiences a high degree of sensory sensitivity. These difficulties are common in people with a diagnosis of ASD.

ASD is a spectrum condition, and Mr Parsons is considered Level 2 out of 3 on the spectrum, according to the Diagnostic and Statistical Manual of Mental Disorders (DSM-5; APA 2013). This is because his symptoms and behaviours affect his everyday functioning, his repetitive behaviours are obvious to the casual observer, he finds it difficult to cope with change, his speech is limited to short sentences and he has a narrow range of specialist interests.

## Appendix 2: Case Vignette

This study concerns a 27-year-old male defendant, in court on a charge of assault and battery of a police officer. Full name: Mr. David Alan Parsons.

### Case Summary

At 11:02 on the 12th June 2017, police were dispatched to Waddon train station in South London regarding reports of an individual acting aggressively on a platform and disturbing the peace. DI Dawes and PC Lovett were notified via radio by central dispatch of a 999 call from the station. The officers were informed by dispatch that according to witnesses the defendant might be aggressive or under the influence of drugs or alcohol.

When the officers arrived on the scene, the defendant—Mr Parsons—was extremely agitated, out of breath and seemed quite distressed. The officers felt that he presented a danger to himself and/or others. They tried to restrain him using handcuffs, which resulted in him becoming verbally and physically aggressive, hitting one of the police officers across the face.

Mr Parsons was detained in a custody suite at the police station where he became increasingly aggressive and abusive towards staff. He was clearly very distressed in custody—visibly sweating and repeatedly rocking back and forth, before he began punching the door. Following this, he was restrained by officers until a healthcare professional (HCP) was available to assess and monitor their behaviour. Mr Parsons was interviewed the next day, following a full risk assessment. During interview, he reported that he was at the station trainspotting, but the train that he was interested in was cancelled, which upset him.

Mr. Parsons lives with his parents and has a job as a cleaner at the local primary school. Clinical assessment found his IQ is within the normal range.

### At Court

In court, Mr Parsons showed little compassion and stared at the ground throughout, avoiding eye contact with the judge and lawyers. During questioning he spoke rapidly whilst rocking back and forth. The following transcript is a brief excerpt from his testimony.

- Lawyer: What were you doing on the 12th of June 2017?
- Defendant: I woke up at 10 o’clock. I left the house at 10:30. I went to get a coffee and a pastry from Starbucks, although the coffee there is shit---.
- Lawyer: *interrupting* We don’t need to know what you had for breakfast Mr. Parsons, and please mind your language. What were you doing at the train station on the 12th June 2017?
- Defendant: Actually, can you not stand so close, your aftershave is really strong, I don’t like it.
- Lawyer: Please just answer the question Mr. Parsons. What were you doing at the train station?
- Defendant: I was watching the trains. I watch the trains every Monday.
- Lawyer: Why did you become aggressive and start swearing at passers-by?
- Defendant: I don’t know. The train was fucking late.
- Lawyer: Do you remember hitting the victim, DI Dawes?
- Defendant: Not really, it was so long ago ... I don’t remember. I didn’t mean to do it.
- Lawyer: So, you accept that you did hit the victim?
- Defendant: I didn’t mean to do it.

## Footnotes

1 It should be noted that offending is nevertheless relatively rare among the autistic population, with several studies showing that autistic individuals are generally as (if not more) law abiding than the general population (Bjørkly 2009; King and Murphy 2014; Lundström et al. 2014; Mouridsen et al. 2008; Woodbury-Smith et al. 2006). Indeed, some have suggested that rigid adherence to rules results in decreased rates of certain types of offending such as property crime, probation offences and those involving pre-meditation (Cheely et al. 2012; Howlin 2004; Kumagami and Matsuura 2009; Woodbury-Smith et al. 2006; but see; Helverschou et al. 2015).
